# FAM65B Regulates Proliferation, Cell Junctions, and Cytoskeletal Organization in Chicken Sertoli Cells

**DOI:** 10.3390/cells15211943

**Published:** 2026-10-26

**Authors:** Jingjing Li, Chuntao Su, Xuliang Luo, Zhen Zhang, Yi Xu, Yanping Feng

**Affiliations:** Key Laboratory of Agricultural Animal Genetics, Breeding and Reproduction of Ministry of Education, College of Animal Science and Technology and College of Veterinary Medicine, Huazhong Agricultural University, Wuhan 430070, China

**Keywords:** FAM65B, Sertoli cells, cell proliferation, cell junctions, cytoskeleton

## Abstract

**Highlights:**

**What are the main findings?**

- FAM65B is highly expressed in rooster testes with high semen quality and specifically localized to cytoplasm of chicken Sertoli cells.
- FAM65B positively regulates Sertoli cell proliferation, cell junction and cytoskeletal remodeling.
- FAM65B knockdown downregulates PI3K-Akt/NF-κB pathways.

**What are the implications of the main findings?**

- FAM65B is a potential regulator of Sertoli cell function in avian spermatogenesis.
- FAM65B expression is associated with semen quality traits in roosters, warranting further investigation.

**Abstract:**

Semen quality in roosters is a critical reproductive trait in poultry, yet its genetic regulatory mechanisms remain poorly understood. Sertoli cells (SCs) are essential for establishing the blood–testis barrier and supporting spermatogenesis. Our previous single-cell transcriptomic analysis identified FAM65B (Family with sequence similarity 65, member B) as a SC-specifically enriched gene that is significantly upregulated in testes of roosters with high semen quality. Given its known roles in cell proliferation, adhesion, and cytoskeletal remodeling, we systematically investigated the spatiotemporal expression pattern of FAM65B in chicken testes and its functional roles in SC physiology using a combination of in vitro and tissue-based approaches. Our results demonstrate that FAM65B is predominantly localized in the SC cytoplasm. Transcriptomic analysis revealed that FAM65B knockdown downregulated genes involved in cell adhesion, cytokine signaling, and the PI3K-Akt and NF-κB pathways. Knockdown of FAM65B significantly inhibited SC proliferation, reduced the expression of junction-associated molecules, and impaired cytoskeletal rearrangement, while its overexpression promoted proliferation, enhanced F-actin filamentous structures, and strengthened cell–cell contacts. Collectively, our findings establish FAM65B as a novel positive regulator of SC function in chickens. These cellular findings, together with the association between FAM65B expression and semen quality traits, provide a foundation for further investigation of SC biology in avian male reproduction.

## 1. Introduction

In poultry production, semen quality of roosters is a primary determinant of fertility, hatchability, and subsequent chick quality. Biologically, the quality of semen is fundamentally dependent on the proper and continuous procession of spermatogenesis within the testes. In testes, the Sertoli cell (SC) is the only somatic cell in direct contact with germ cells. SCs provide not only physical support and nutrients but also create a unique immune-privileged microenvironment via the blood–testis barrier (BTB), and orchestrate spermatogenesis through the secretion of growth factors that govern the fate of spermatogonial stem cells [1,2,3]. However, the molecular mechanisms governing SC function in poultry, particularly in chickens, remain largely unexplored. This knowledge gap hinders the development of targeted strategies to address reproductive inefficiencies in rooster breeding.

The integrity of the BTB, formed by tight junctions, adherens junctions, and a robust cytoskeletal network between adjacent SCs, is essential for spermatogenesis. Disruption of this barrier is a direct cause of spermatogenic failure [2]. Studies in mammals have demonstrated that deletion of key SC-specific genes can lead to disruption of the BTB and male infertility. For instance, conditional knockout of hnRNPH1 impairs BTB function and meiosis, leading to male infertility in mice [4]. Similarly, RNF20 regulates the transcription of tight junction gene Cldn11 via histone ubiquitination, and its deficiency leads to disorganization of the seminiferous tubules and abnormal apoptosis of spermatogenic cells [5]. Smtnl2, an actin-binding protein, maintains cytoskeletal stability and BTB integrity; its knockout results in germ cell sloughing and altered lactate metabolism [6]. In contrast, research on rooster fertility has largely been confined to the identification of candidate genes through transcriptomic screenings, with limited functional validation in avian SCs.

Our preliminary single-cell RNA-sequencing (scRNA-seq) of chicken testes revealed FAM65B (Family with sequence similarity 65, member B) as a SC-specific gene with significantly higher expression in roosters with superior semen quality. FAM65B is an atypical inhibitor of the small GTPase RhoA and a transcriptional target of FOXO1 [7]. It participates in several cytoskeleton-dependent processes, including T-cell cycle regulation [8,9], neutrophil adhesion and polarization [10], and myoblast migration and fusion [11]. Notably, RhoA signaling is known to crosstalk with both the PI3K-Akt pathway, which promotes cell proliferation and survival [12,13], and the NF-κB pathway, which regulates diverse cellular responses in a context-dependent manner [14,15]. Given that FAM65B functions as a RhoA inhibitor, it may indirectly influence these pathways in SCs. Importantly, it has been well established that both the RhoA signaling pathway and the FOXO1 transcription factor play critical roles in mammalian spermatogenesis. Disruption of RhoA signaling compromises BTB integrity and spermatid adhesion [16,17], while aberrant FOXO1 activation is linked to germ cell apoptosis and spermatogenic damage [18]. Given these connections and our preliminary data, we hypothesized that FAM65B is a key regulator of SC proliferation and cell junctions, thereby influencing spermatogenesis and semen quality traits in roosters.

In this study, we systematically characterized the expression pattern of FAM65B in chicken testes and investigated its functional role in primary SCs through gain- and loss-of-function experiments. Our results elucidate the mechanistic roles of FAM65B in regulating cell proliferation, adhesion, tight junction, and cytoskeletal reorganization—all of which are critical for spermatogenesis. Together, these findings reveal FAM65B as a novel regulator of SC function in avian testes and provide a foundation for further investigating its causal relationship with semen quality traits in roosters.

## 2. Materials and Methods

### 2.1. Animals and Sample Collection

Wenchang roosters (1-day-old) were obtained from Hainan (Tanniu) Wenchang Chicken Co., Ltd. (Wenchang, China) and raised in the experimental farm of Huazhong Agricultural University. At 35 weeks of age, semen volume was measured with pipette, and semen parameters were determined by computer assisted analysis (CASA, Minitube AndroVision, Minitube, Germany) after dilution the semen with physiological saline at a ratio of 1:80 (*v*/*v*). The semen quality assessment was completed within 15 min, with 3 ejaculates collected per rooster for the measurements of each indicator. Based on the combined parameters scores of semen volume, sperm concentration and total motility, individuals with the highest and lowest semen quality were selected as the high-quality (CH) and low-quality (CL) groups (*n* = 4 per group). Testes from 1-day chicken to 36-week rooster and other tissues (hypothalamus, pituitary, thyroid, heart, liver, lung, kidney, and muscle) of 36-week rooster were collected post-euthanised by carotid artery bleeding after Deep anesthesia. One portion of testis was immediately frozen in liquid nitrogen for RNA extraction, and another was fixed in 4% paraformaldehyde (PFA) for histological analysis.

### 2.2. Primary Gonadal Sertoli Cell Isolation and Culture

Testes were aseptically collected from 18-day-old chicken embryos (Jingfen No.1, Hubei Yukou Poultry Co., Ltd. from Jingzhou, China). The dissected testes were rinsed twice with PBS, and blood vessels and adherent connective tissues were removed with forceps. The cleaned testes were minced into small pieces with ophthalmic scissors, then digested with 10 vollumes of 0.25% trypsin-EDTA at 37 °C for 10 min. After centrifugation (800 rpm, 3 min), trypsin was removed and an equal volume of 1 mg/mL collagenase was added at 37 °C for 20 min. Digestion was stopped with DMEM (Gibco, Waltham, MA, USA) supplemented with 10% fetal bovine serum (FBS, Gibco) and 1% penicillin-streptomycin, and cell suspension was filtered through a 40 µm cell strainer, centrifuged, and resuspended in fresh DMEM containing 10% FBS. Cells were seeded into culture plates and incubated at 37 °C in a 5% CO_2_ atmosphere. For immunofluorescence, cells were cultured on sterile glass coverslips.

### 2.3. Gene Knockdown and Overexpression in Sertoli Cells

Small interfering RNAs (siRNAs) targeting chicken FAM65B (Gene ID: 421003) and a negative control (si-NC) were designed and synthesized by GenePharma (Suzhou, China, sequences shown in Table A1). For overexpression, the coding sequence (CDS) of FAM65B was amplified and cloned into the pcDNA3.1(+) vector (with BamHI and EcoRI sites) by Hippo Biotech (Huzhou, China, primer sequences shown in Table A1). SCs at 70–80% confluence were transfected with siRNAs or plasmids using JetPRIME^®^ transfection reagent (Polyplus-transfection, Illkirch, France) according to the manufacturer’s protocol. Cells were harvested 24–48 h post-transfection for RNA, protein, or functional assays. All experiments included four biological replicates per group.

### 2.4. RNA-Sequencing and Differential Expression Analysis

Total RNA was extracted from SCs transfected with si-FAM65B or si-NC. Libraries were sequenced on an Illumina HiSeq X-Ten platform (Bioyigene, Wuhan, China). Differentially expressed genes (DEGs) were identified using a threshold of |log2(Fold Change)| ≥ 1 and padj ≤ 0.05. Gene Ontology (GO) and Kyoto Encyclopedia of Genes and Genomes (KEGG) pathway enrichment analyses were performed using the clusterProfiler package in R. Gene Set Enrichment Analysis (GSEA) was also conducted. The RNA-seq data were analyzed on the Bioyigene Cloud Platform (https://cloud.bioyigene.com/).

### 2.5. Immunohistochemistry (IHC) and Immunofluorescence (IF)

Fixed testis tissues were embedded in paraffin, sectioned (5 µm), and deparaffinized. For IHC, antigen retrieval was performed in 0.01 M sodium citrate buffer. Sections were blocked with 5% BSA and incubated with primary antibodies against FAM65B (A9234, ABclonal, 1:500) and HRP-conjugated secondary antibodies. DAB was used for color development, and sections were counterstained with hematoxylin. For IF, after primary antibody incubation, sections were incubated with a fluorescent secondary antibody. For co-localization, sections were sequentially stained with antibodies against FAM65B and SOX9 (ab185230, Abcam, 1:500), followed by appropriate fluorophore-conjugated secondary antibodies. Nuclei were counterstained with DAPI, and images were captured using a fluorescence microscope (Olympus BX63, Tokyo, Japan).

### 2.6. RNA Extraction and Quantitative Real-Time PCR (qPCR)

Total RNA was extracted from cells and tissues using TRIzol reagent (Invitrogen, Carlsbad, CA, USA) and reverse-transcribed using HiScript II Q RT SuperMix (Vazyme, Nanjing, China). qPCR was performed on a CFX-384 Real-Time PCR System (Bio-Rad, Hercules, CA, USA) using QuantiFast SYBR Green PCR Master Mix (Qiagen, Hilden, Germany). The GAPDH gene was used as an internal control. Relative expression was calculated using the 2^−ΔΔCT^ method. All primer sequences are listed in Table A2.

### 2.7. Western Blotting

SCs were lysed in RIPA buffer containing protease and phosphatase inhibitors. Proteins were separated by SDS-PAGE, transferred to PVDF membranes, and blocked with 5% BSA. Membranes were incubated overnight at 4 °C with primary antibodies against PCNA (A0264, ABclonal), β-catenin (ab16051, Abcam), ZO-1 (ab216880, Abcam), β-Tubulin (A12289, ABclonal), GAPDH (A10868, ABclonal) and F-actin (ab130935, Abcam), followed by HRP-conjugated secondary antibodies. Immunoreactive bands were visualized using an ECL chemiluminescence kit (Yakoyin Biotech, Wuhan, China) and quantified with ImageJ 1.53e software.

### 2.8. EdU Incorporation Assay

Cell proliferation was assessed using the EdU Cell Proliferation Kit (Yakoyin Biotech) according to the manufacturer’s instructions. SCs were incubated with 10 µM EdU for 2 h. After fixation and permeabilization, EdU was detected using a Click-iT reaction cocktail. Nuclei were stained with DAPI. The percentage of EdU-positive cells (proliferating cells) was calculated from images captured by a fluorescence microscope (Olympus BX63) using Image Pro Plus 6.0 software.

### 2.9. F-Actin Staining

To visualize the cytoskeleton, SCs cultured on coverslips were fixed with 4% PFA 15 min, permeabilized with 0.1% Triton X-100 10 min, and blocked with 5% BSA 1 h. Filamentous actin (F-actin) was stained with 200 μL/well rhodamine-conjugated phalloidin (Yeasen, Shanghai, China), and nuclei were counterstained with DAPI. Stained cells were examined and imaged using a Nikon AXR NSPARC confocal microscope (Tokyo, Japan).

### 2.10. Statistical Analysis

All experiments data were presented as mean ± standard deviation (SD). Statistical analyses were performed using GraphPad Prism 8. Comparisons between two groups were conducted using a two-tailed Student’s *t*-test, and among multiple groups using one-way ANOVA for normally distributed data or the Kruskal–Wallis test for non-normally distributed data. A *p*-value < 0.05 was considered statistically significant (* *p* < 0.05, ** *p* < 0.01, *** *p* < 0.001).

## 3. Results

### 3.1. Spatiotemporal Expression and Cellular Localization of FAM65B in Rooster Testes

**Figure 1:**
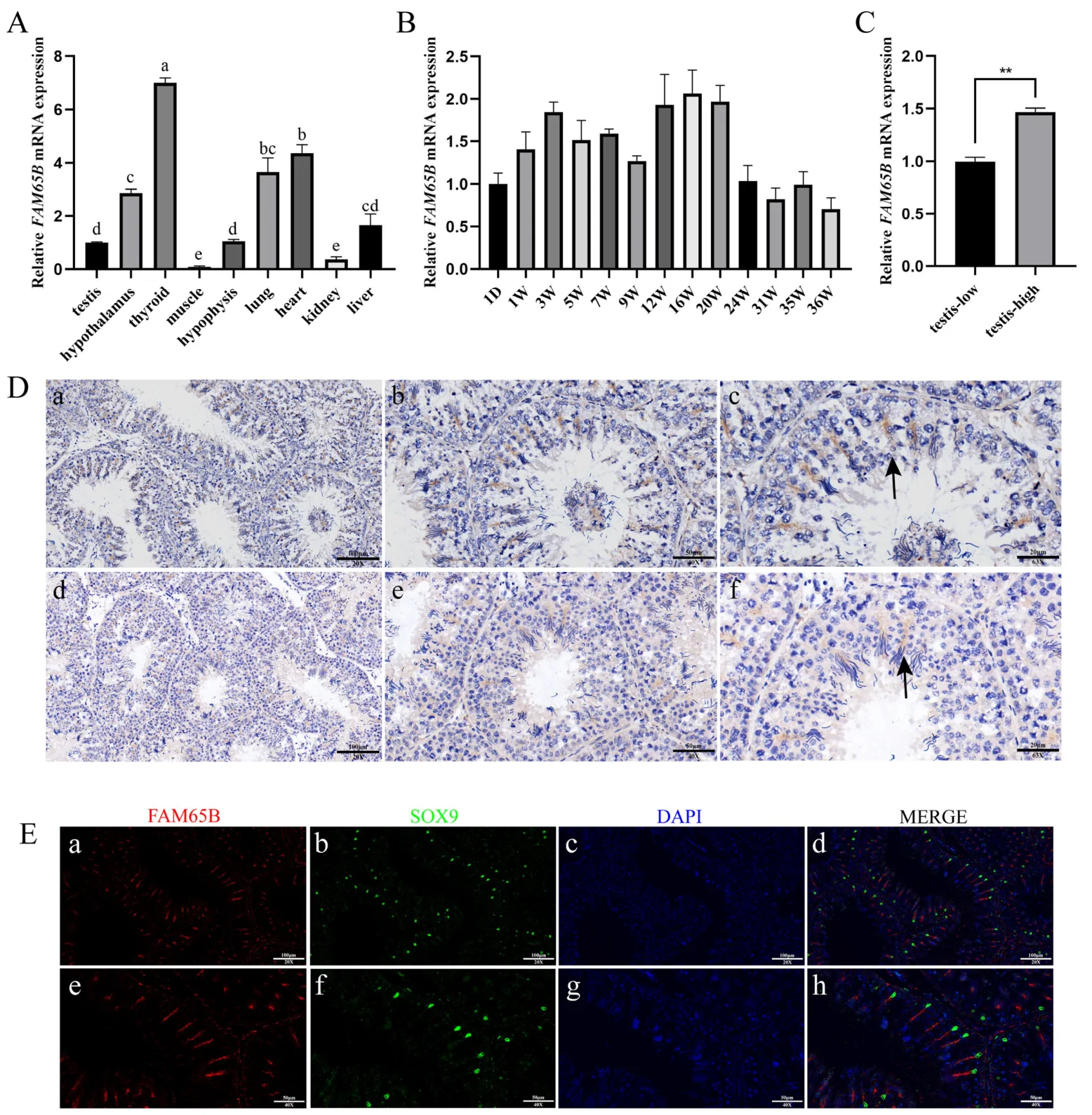
Expression and localization of FAM65B in rooster tissues and testes. (**A**) Relative mRNA expression of FAM65B in various tissues from 35-week-old roosters (*n* = 3). Different letters above the bars indicate significant differences among groups (*p* < 0.05). (**B**) Temporal expression profile of FAM65B in chicken testes from 1 day to 36 weeks of age (*n* = 3). D, days; W, weeks. (**C**) qPCR showing significantly higher FAM65B expression in high vs. low semen quality groups (*n* = 4). ** *p* < 0.01. (**D**) Immunohistochemical staining showing FAM65B (brown) in the seminiferous epithelium. (a–c) High semen quality group; (d–f) Low semen quality group; (a,d) 20× magnification; (b,e) 40× magnification; (c,f) 63× magnification. (**E**) Double immunofluorescence staining showing co-localization of FAM65B (red) and SOX9 (green) in SCs. (a–d) 20× magnification; (e–h) 40× magnification.

To explore the potential role of FAM65B in spermatogenesis, its expression profiles were firstly determined in various tissues and testes at different developmental stages. qPCR analysis in 36-week-old roosters revealed that FAM65B was broadly expressed across multiple tissues, with the highest levels detected in the thyroid. Notably, moderate expression was also observed in the testis (Figure 1A). In the testis, its expression exhibited two peaks: the first at 3 weeks of age and the second between 12 and 20 weeks of age (Figure 1B), corresponding to active SC proliferation and the onset of sexual maturity, respectively. Importantly, both qPCR and IHC analyses confirmed that FAM65B expression was significantly higher in the testes of the high semen quality (CH) group than in the low semen quality (CL) group (Figure 1C,D). IHC showed that FAM65B protein was distributed in a bundled pattern across the seminiferous epithelium (Figure 1D). Double immunofluorescence (IF) staining demonstrated that FAM65B co-localized with SOX9 (a SC marker) in the testis, and was expressed in the SC cytoplasm, corresponding to but not overlapping with SOX9, which was localized in the SC nuclei (Figure 1E).

### 3.2. Transcriptomic Analysis of SCs Following FAM65B Knockdown

**Figure 2:**
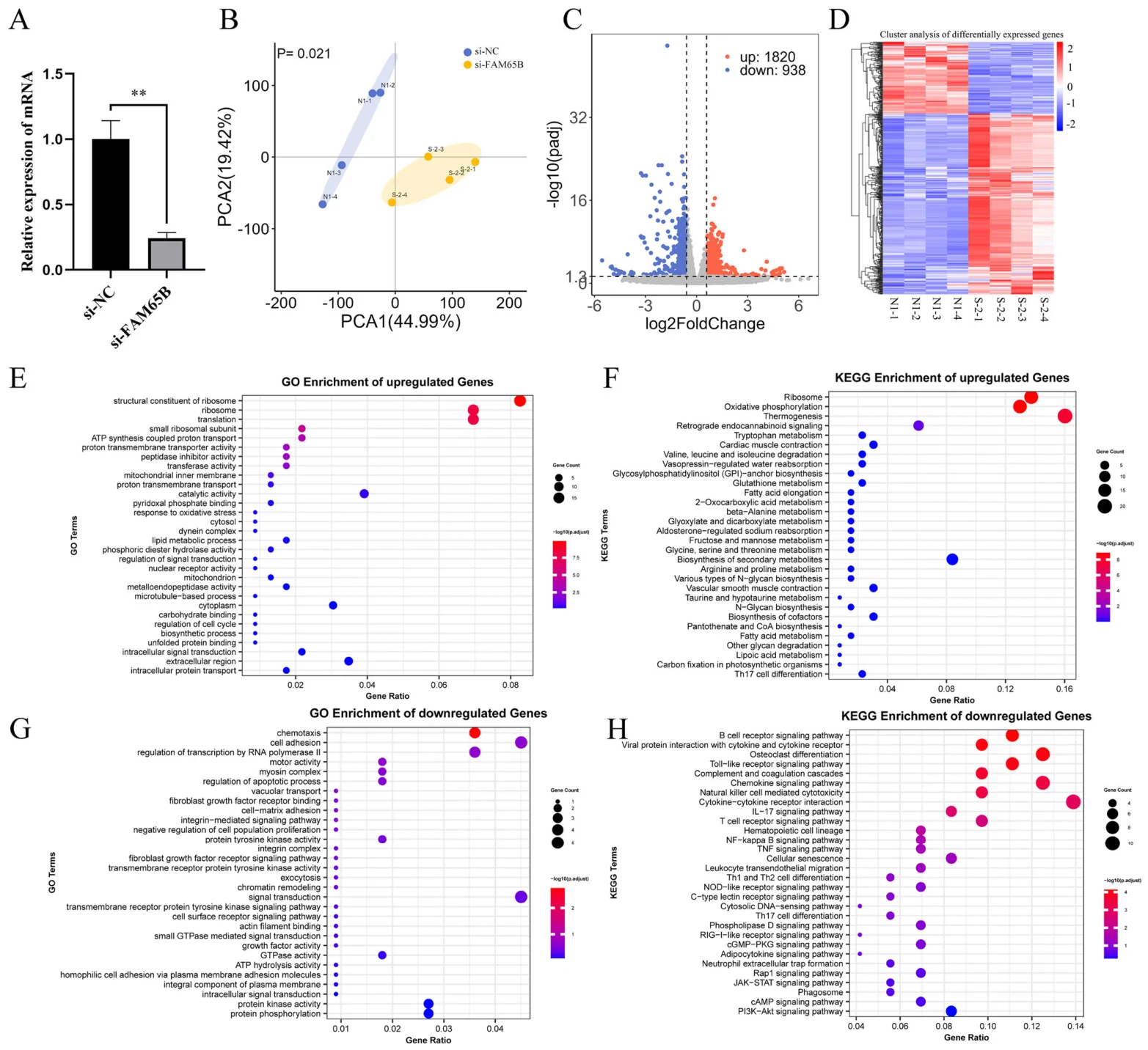
Transcriptomic analysis of Sertoli cells after FAM65B knockdown. (**A**) qPCR validation of FAM65B knockdown efficiency at 24 h post-transfection with si-FAM65B (*n* = 4). ** *p* < 0.01. (**B**) PCA plot showing distinct clustering of si-NC and si-FAM65B groups. (**C**) Volcano plot of DEGs (si-FAM65B vs. si-NC). (**D**) Heatmap of DEGs. N1, si-NC control group; S-2, si-FAM65B knockdown group. (**E**,**G**) GO enrichment analysis of upregulated (**E**) and downregulated (**G**) DEGs. (**F**,**H**) KEGG pathway enrichment analysis of upregulated (**F**) and downregulated (**H**) DEGs.

**Figure 3:**
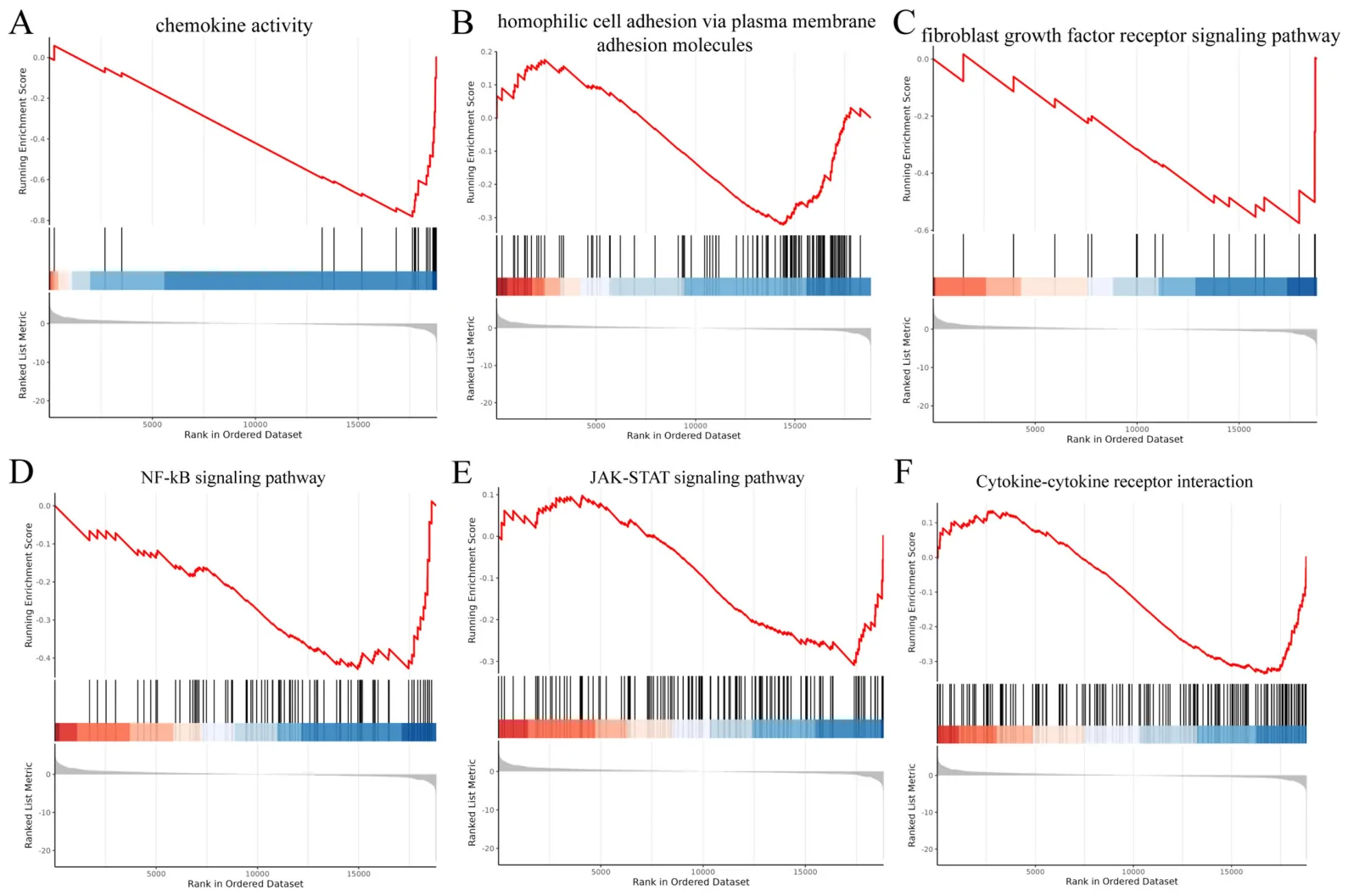
GSEA of downregulated DEGs in FAM65B-knockdown Sertoli Cells. GSEA plots showing significant downregulation of gene sets related to (**A**) chemokine activity, (**B**) homophilic cell adhesion via plasma membrane adhesion molecules, (**C**) fibroblast growth factor receptor signaling pathway, (**D**) NF-k B signaling pathway, (**E**) JAK-STAT signaling pathway, and (**F**) cytokine–cytokine receptor interaction.

**Figure A1:**
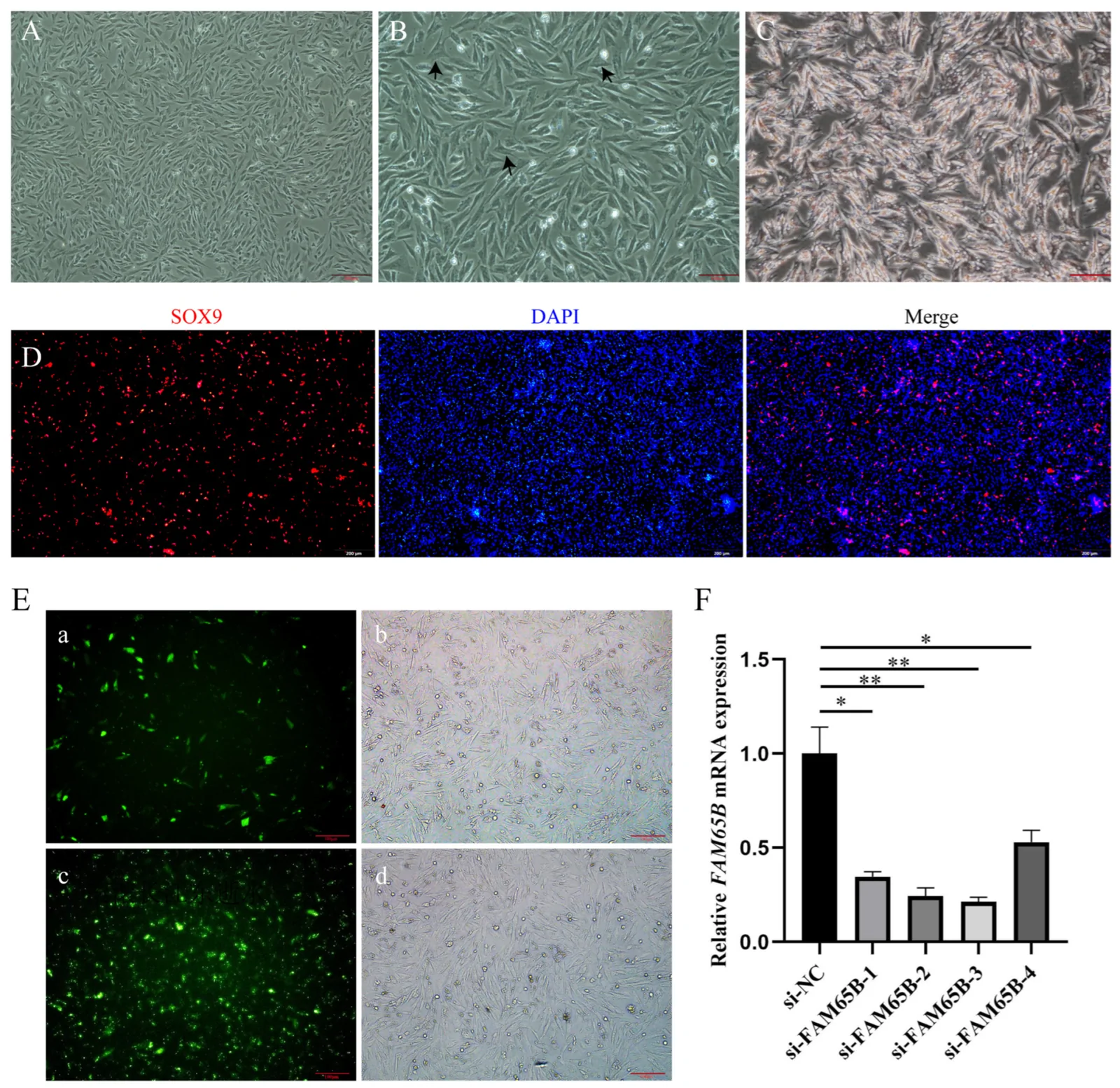
FAM65B knockdown in chicken Sertoli cells and assessment of its effects. (**A**,**B**) Sertoli cells culture, which photos in the open field with magnification of 10× (**A**) and magnification of 20× ((**B**), arrows show the polysome structure of Sertoli cells); (**C**) Oil red O staining of sertoli cells with magnification of 20×; (**D**) Immunofluorescence identification of sertoli cells (red, SOX9; blue, DAPI nuclear staining); (**E**) Fluorescence rendering of FAM65B transfected sertoli cells (a,b) were fluorescence and bright field images after transfection for 6 h, respectively. (c,d) were fluorescence and bright field images after transfection for 24 h, respectively. (**F**) Relative expression of FAM65B in sertoli cells after transfection with 4 siRNAs, *n* = 3; *, *p* < 0.05; **, *p* < 0.01.

To delineate the functional landscape of FAM65B in SCs, we performed RNA-seq on SCs transfected with si-FAM65B or si-NC. Efficient knockdown was confirmed by qPCR (Figure 2A, Figure A1). Principal component analysis (PCA) and clustering analysis showed clear separation between the si-FAM65B and si-NC groups, indicating robust transcriptomic changes (Figure 2B,D). A total of 1820 upregulated and 938 downregulated DEGs were identified in the si-FAM65B group (Figure 2C). GO and KEGG enrichment analyses revealed that upregulated genes were enriched in ribosomal structure, translation, and oxidative phosphorylation (Figure 2E,F). In contrast, downregulated genes were significantly enriched in biological processes and pathways related to cell adhesion, chemotaxis, cytokine–cytokine receptor interaction, NF-κB, and PI3K-Akt signaling (Figure 2G,H), suggesting a central role for FAM65B in these critical SC functions. GSEA further confirmed the significant downregulation of gene sets associated with chemokine activity, fibroblast growth factor receptor binding, homophilic cell adhesion, NF-κB, and JAK-STAT signaling pathways (Figure 3A–F).

### 3.3. FAM65B Regulates Sertoli Cell Proliferation

**Figure 4:**
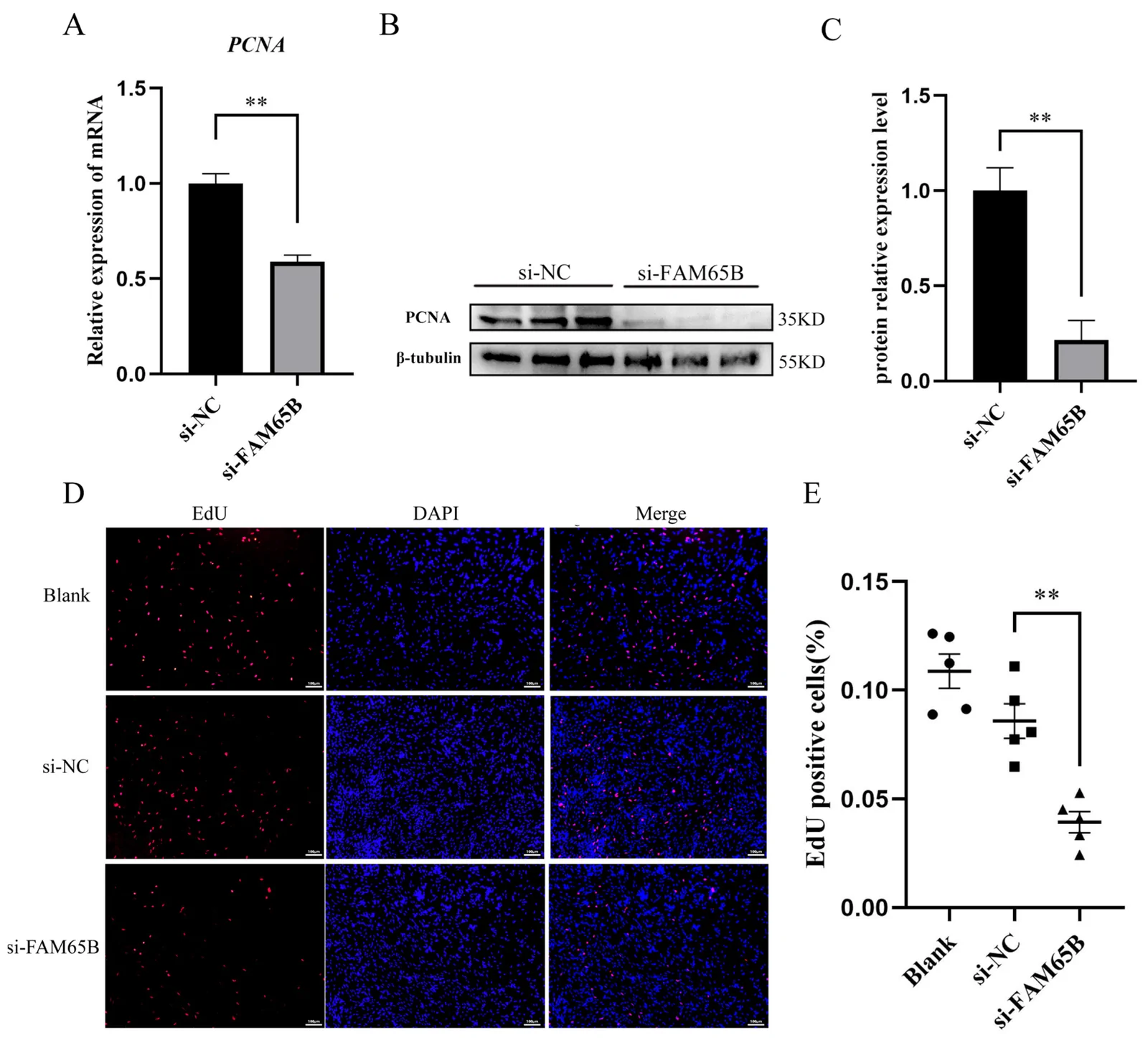
FAM65B knockdown inhibits Sertoli cell proliferation. (**A**) Relative mRNA expression of PCNA in SCs transfected with si-FAM65B or si-NC, as determined by qPCR. (**B**) Western blot analysis of PCNA protein expression in SCs following FAM65B knockdown. (**C**) Relative protein levels of PCNA normalized to β-tubulin. (**D**) EdU incorporation assay in SCs after FAM65B knockdown (red, EdU-positive cells). Scale bar = 100 µm (20× magnification). (**E**) Percentage of EdU-positive SCs (*n* = 5). Data are presented as mean ± SD. ** *p* < 0.01.

**Figure 5:**
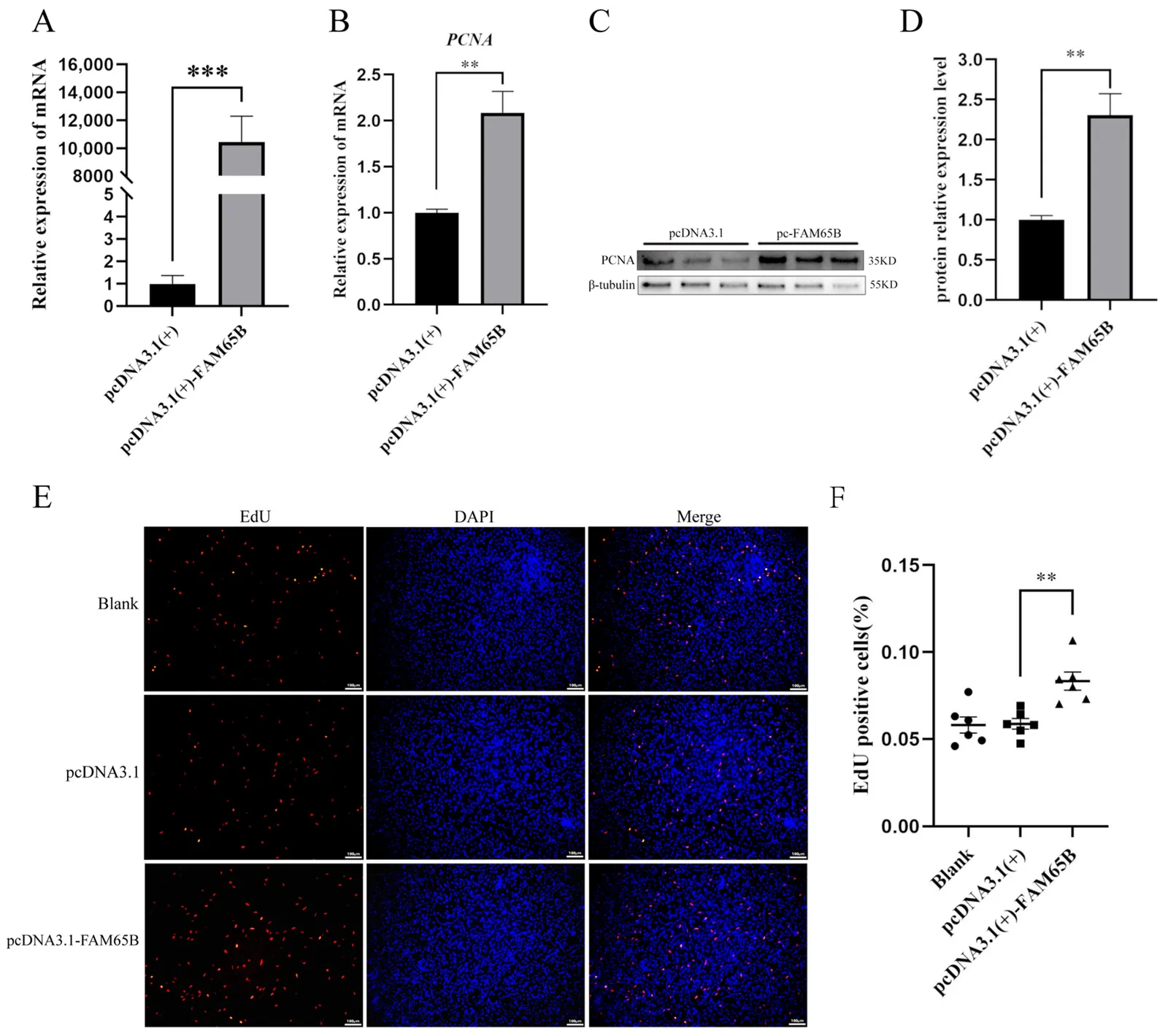
FAM65B overexpression promotes Sertoli cell proliferation. (**A**) qPCR validation of FAM65B overexpression 24 h post-transfection. (**B**) Relative mRNA expression of PCNA in SCs following FAM65B overexpression, detected by qPCR. (**C**) Western blot analysis of PCNA protein expression in SCs after FAM65B overexpression. (**D**) Relative protein levels of PCNA normalized to β-tubulin. (**E**) EdU incorporation assay in SCs after FAM65B overexpression (red, EdU-positive cells). Scale bar = 100 µm (20× magnification). (**F**) Percentage of EdU-positive SCs (*n* = 6). Data are presented as mean ± SD. ** *p* < 0.01, *** *p* < 0.001.

Given the enrichment of downregulated DEGs in proliferative signaling pathways, with a marked decrease in the expression of the proliferation marker gene PCNA (*p* < 0.001) (Supplementary Table), both loss-of-function and gain-of-function experiments were performed to investigate the effect of FAM65B on SC proliferation. qPCR and Western blot (WB) analyses confirmed a significant decrease in the expression of the proliferation marker gene PCNA at both the mRNA and protein levels in si-FAM65B-treated SCs (Figure 4A–C). Consistently, EdU incorporation assays showed a significant reduction in the percentage of proliferating cells (EdU-positive) in the si-FAM65B group compared to controls (Figure 4D,E). Conversely, overexpression of FAM65B via pcDNA3.1-FAM65B transfection led to significantly elevated PCNA expression at both the mRNA and protein levels (*p* < 0.01) (Figure 5B–D) and a significant increase in EdU-positive cells (*p* < 0.01) (Figure 5E,F). Collectively, these results demonstrate that FAM65B positively regulates SC proliferation in chickens.

### 3.4. FAM65B Modulates Cell Junction Integrity and Cytoskeletal Organization in SCs

**Figure 6:**
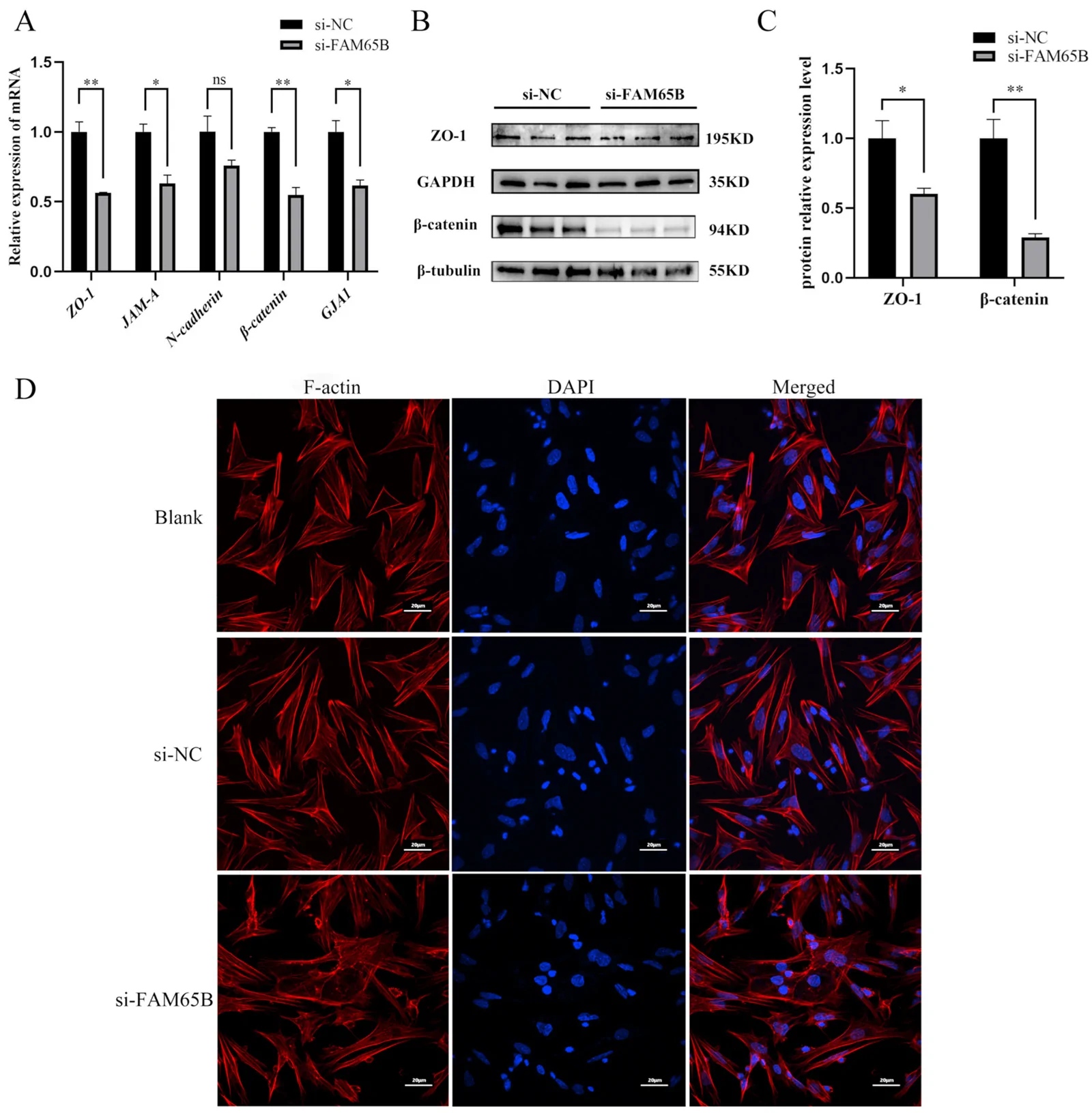
FAM65B knockdown disrupts cell junctions and cytoskeletal organization. (**A**) qPCR analysis of junction-related genes (ZO-1, JAM-A, N-cadherin, β-catenin, GJA1) in SCs after FAM65B knockdown. (**B**) Western blot analysis of ZO-1 and β-catenin protein expression in SCs after FAM65B knockdown. (**C**) Relative protein levels of ZO-1 and β-catenin normalized to β-tubulin. (**D**) Phalloidin staining (red) of F-actin showing disorganized cytoskeleton in FAM65B-knockdown SCs; DAPI (blue) stains nuclei. Scale bar = 20 µm (63× magnification). Data are presented as mean ± SD (*n* = 3). ns, not significant; * *p* < 0.05, ** *p* < 0.01.

**Figure 7:**
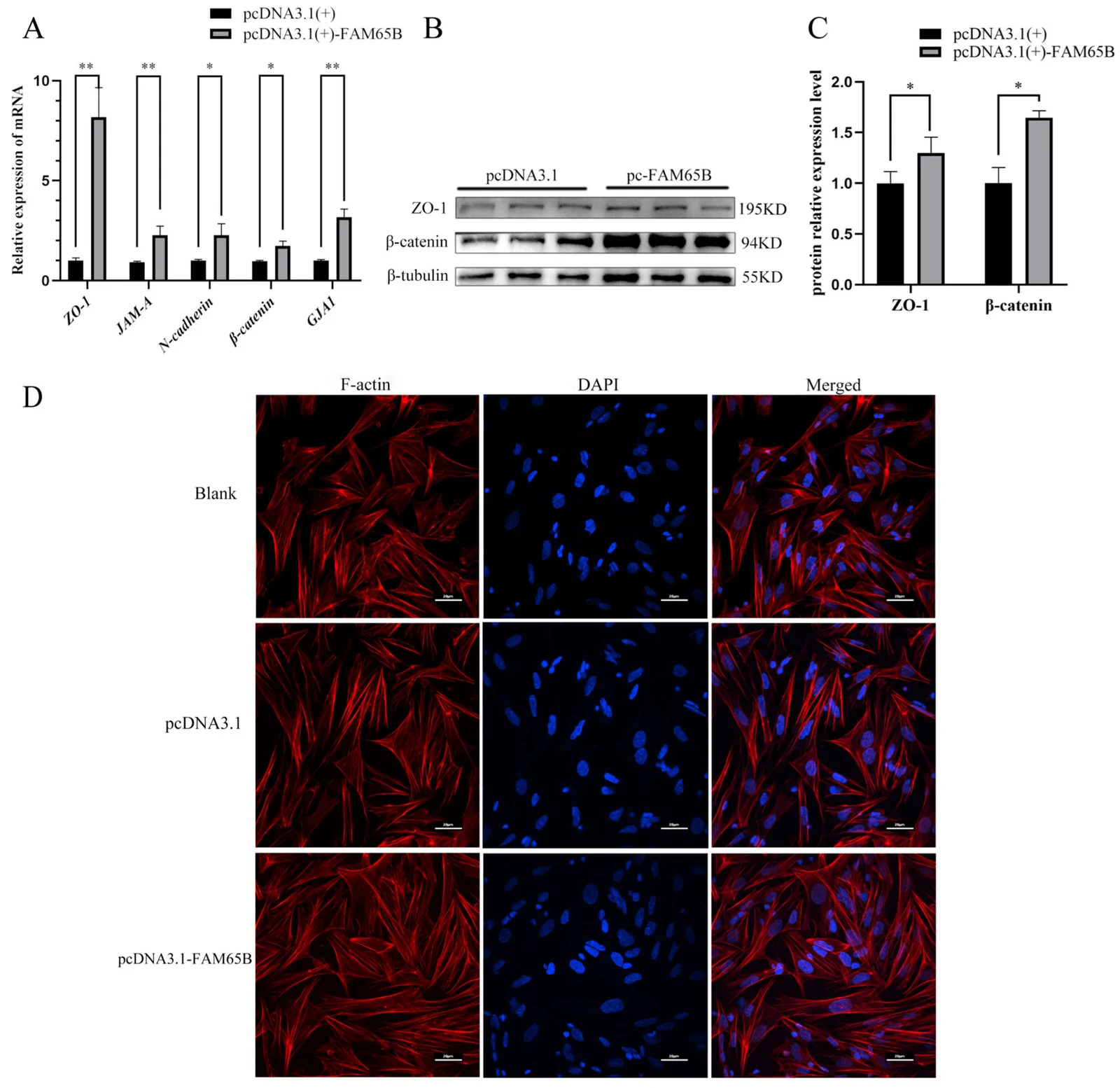
FAM65B overexpression enhances cell junctions and cytoskeletal organization. (**A**) qPCR analysis of junction-related genes in SCs overexpressing FAM65B. (**B**) Western blot analysis showing increased ZO-1 and β-catenin protein levels. (**C**) Relative protein levels of ZO-1 and β-catenin normalized to β-tubulin. (**D**) Phalloidin staining (red) of F-actin showing a more robust and organized cytoskeleton and enhanced cell–cell contacts in FAM65B-overexpressing SCs; DAPI (blue) stains nuclei. Scale bar = 20 µm (63× magnification). Data are mean ± SD (*n* = 3). * *p* < 0.05, ** *p* < 0.01.

Based on the RNA-seq data suggesting a role for FAM65B in cell adhesion and cytoskeleton dynamics, the expression of key junctional proteins in SCs were examined subjected to either FAM65B knockdown or overexpression. qPCR revealed that the mRNA levels of ZO-1, JAM-A, β-catenin, and GJA1 were significantly reduced upon FAM65B knockdown, while N-cadherin showed no significant change (Figure 6A). Western blotting confirmed a significant decrease in the protein levels of ZO-1 and β-catenin in si-FAM65B SCs (Figure 6B,C). Furthermore, phalloidin staining of F-actin revealed that, in contrast to the well-organized stress fibers and clear cell–cell contacts in control cells, FAM65B-knockdown SCs displayed a disorganized cytoskeleton with irregular, fragmented stress fibers and less compact cell–cell contacts (Figure 6D). Concordantly, overexpression of FAM65B significantly upregulated the expression of junction-related genes (ZO-1, JAM-A, N-cadherin, β-catenin, GJA1) at the mRNA level (Figure 7A) and increased the protein levels of ZO-1 and β-catenin (Figure 7B, C). Most strikingly, phalloidin staining revealed that FAM65B-overexpressing SCs exhibited a more robust and organized F-actin network with prominent filamentous structures and well-defined, compact cell–cell contacts compared to controls (Figure 7D). These findings demonstrate that FAM65B positively regulates cell junction integrity and cytoskeletal organization in chicken SCs.

## 4. Discussion

This study provides the first functional evidence that FAM65B is a critical regulator of avian spermatogenesis. We demonstrate that FAM65B, a cytoplasmically localized protein specifically expressed in chicken Sertoli calls, positively regulate SC proliferation, cell junction formation, and cytoskeletal dynamics. Its dynamic expression during testis maturation, together with its elevated levels in roosters with higher semen quality, strongly supports it as a promising candidate for male fertility in poultry.

The expression pattern of FAM65B in the developing chicken testis suggests its involvement in distinct phases of SC function. The first peak at 3 weeks of age coincides with the rapid proliferative phase of SCs, which determines the final SC number and, consequently, the sperm-producing capacity of the testis [19,20]. The second, more pronounced peak at 12–20 weeks corresponds to the onset of sexual maturity, when SCs establish and maintain a mature BTB and support meiosis and spermiogenesis. This biphasic expression pattern suggests a dual role for FAM65B: first in expanding the SC population, and later in maintaining SC function to support germ cell development. Importantly, this notion is directly supported by our functional data. RNA-seq analysis of FAM65B knockdown SCs revealed significant downregulation of PI3K-Akt, NF-κB signaling pathways, both of which are known to be crucial for SC proliferation and survival [21,22,23]. Consistently, our loss- and gain-of-function experiments demonstrated that FAM65B positively regulates SC proliferation, as evidenced by altered PCNA expression and EdU incorporation. Thus, the coordinated expression and functional data establish FAM65B as a key player in SC proliferation, with its role extending into the post-pubertal testis.

Beyond its role in proliferation, our results indicate that FAM65B is involved in regulating the expression of junction-associated molecules and cytoskenetal organization in SCs. The BTB and adherens junctions between SCs are essential for forming an impermeable barrier and for the directed migration of developing germ cells from the basal to the adluminal compartment [1,2]. In our study, qPCR and Western blot analyses demonstrated that knockdown of FAM65B significantly reduced the expression of key junctional proteins, including ZO-1 (a tight junction component) and β-catenin (an adherens junction component), whereas overexpression of FAM65B yielded the opposite effects. While these data demonstrate changes in total β-catenin protein levels, they do not address its phosphorylation status. β-catenin function is tightly regulated by site-specific phosphorylation, with distinct residues controlling its degradation (Ser33/37/Thr41/Ser45) [24] versus its junctional and transcriptional activity (Ser552) [25]. Future phosphoproteomic studies identifying FAM65B-regulated β-catenin phosphorylation sites would provide additional mechanistic insight into how FAM65B modulates junctional stability in avian Sertoli cells.

Furthermore, phalloidin staining of F-actin revealed that FAM65B knockdown disrupted the organized network of stress fibers and led to looser cell–cell contacts, while overexpression promoted a more robust and well-organized F-actin cytoskeleton with compact intercellular contacts. These findings are consistent with the known function of FAM65B in modulating RhoA signaling, a master regulator of actin dynamics and cell adhesion [7,10]. Given that RhoA governs actin polymerization and actomyosin contractility, we propose that FAM65B coordinates cytoskeletal remodeling and junctional stability in SCs by fine-tuning RhoA activity. This regulatory mechanism may contribute to the dynamic remodeling of the BTB and the maintenance of SC polarity, both of which are essential for spermatid translocation and germ cell progression. In other models where cell adhesion is compromised, disruption of this balance leads to impaired BTB integrity and germ cells sloughing [26,27], ultimately reducing fertility. However, it should be noted that these data reflect changes in junction-related molecule expression and cytoskeletal organization, rather than a direct assessment of barrier function. F-actin staining was performed on Sertoli cells cultured under proliferative conditions on glass coverslips, rather than on polarized monolayers grown on Matrigel-coated bicameral units, which is the established in vitro system for studying BTB dynamics [28]. Therefore, while our findings are consistent with a potential role for FAM65B in junctional regulation, they do not functionally confirm that barrier integrity is compromised. Future studies employing the established Transwell-based BTB model with transepithelial electrical resistance (TER) measurement are warranted to evaluate the functional impact of FAM65B on barrier integrity.

Our transcriptomic data provide further mechanistic insights into how FAM65B orchestrate SC function. Beyond the downregulation of junction- and cytoskeleton-related genes, RNA-seq analysis revealed that FAM65B knockdown significantly affected genes involved in the NF-κB and PI3K-Akt pathways, providing a plausible molecular explanation for the reduced SC proliferation observed in our loss-of-function experiments. Additionally, the enrichment of cytokine–cytokine receptor signaling and adhesion-related genes sets suggests that FAM65B may also participate in SC-germ cell communication, a process essential for spermatogonial differentiation and meiotic progression. Notably, FAM65B is a known transcriptional target of FOXO1 in immune cells [7], and FOXO1 has been shown to regulate both proliferation and apoptosis in mammalian testis [18]. Taken together with our findings, it is plausible that a similar FOXO1-FAM65B-RhoA operates in chicken SCs, acting as a signaling hub that integrates upstream cues to modulate diverse cellular outcomes, including proliferation, junction stability, and cytoskeletal dynamics. While our current study establishes FAM65B as a key player in avian SC biology, future work will focus on identifying its direct interactors in SCs and dissecting the precise molecular network of this regulatory network.

A further limitation is that our functional experiments used embryonic Sertoli cells cultured adherent conditions. Although these in vitro data demonstrate a regulatory role for FAM65B in Sertoli cell proliferation, junction-related molecule expression, and cytoskeletal organization, they do not establish whether these cellular changes translate into altered semen quality in vivo. Future studies using in vivo knockdown/overexpression of FAM65B in adult roosters determine whether FAM65B functions similarly in the mature testis and to directly evaluate its impact on semen quality and testicular function.

## 5. Conclusions

In conclusion, our study has identified FAM65B as a novel and essential regulator in chicken SC function. FAM65B positively regulates Sertoli cell proliferation, the expression of junction-associated molecules, and cytoskeletal organization. These findings fill a significant gap in our understanding of Sertoli cell biology in avian species and, together with the positive association between FAM65B expression and semen quality traits, provide a foundation for further investigation of its potential role in rooster reproductive performance.

## Data Availability

The original contributions presented in this study are included in the article and Supplementary Material. Further inquiries can be directed to the corresponding author.

## Supplementary Materials

The following supporting information can be downloaded at: https://www.mdpi.com/article/10.3390/cells15211943/s1, Figure S1: Original uncropped Western blot membranes; Table S1: Si-NC_vs_Si-FAM65B Gene expression; Table S2: Si-NC vs_Si-FAM65B Differential analysis results; Table S3: Si-NC_vs_Si-FAM65B DEG Annotation; Table S4: Si-NC_vs_Si-FAM65B GSEA go enrichment.

## Appendix A

**Table A1:** List of siRNA sequences and primers for amplifying FAM65B CDS.

Name	Sequences (5′-3′)
Si-FAM65B-1	GGGAGUCCUGUAUGAUUUATT
	UAAAUCAUACAGGACUCCCTT
Si-FAM65B-2	GCUUUACGAGGCCUACUGUTT
	ACAGUAGGCCUCGUAAAGCTT
Si-FAM65B-3	GAACAUGUGCACCAUAGAATT
	UUCUAUGGUGCACAUGUUCTT
Si-FAM65B-4	GCGUUACCUGUGAAACAAATT
	UUUGUUUCACAGGUAACGCTT
Si-NC	GCGACGAUCUGCCUAAGAU dTdT
	AUCUUAGGCAGAUCGUCGC dTdT
FAM65B CDS	F: cgggatccATGTCAATAGGATCACATTC
	R: cggaattcTCAGATATCCTTTAGTGTAG

Note: Lowercase letters in the primer sequence represent restriction enzyme cutting sites.

**Table A2:** Primer sequences used for qPCR.

Primer Name	Sequences (5′-3′)
FAM65B	GACCTTGGCACAATCAAGCT
	TCTCTGGAGTGCCTTGGCTGT
PCNA	GACAATGCGGATACGTTGGC
	TCACCAATGTGGCTGAGGTC
GJA1	CGGCAAGGTAAAGATGCGTG
	ATGGCACTCAGGCTAAACCC
JAM-A	GTCACCTCGGAGACAAAGGA
	TGTGAGCTCTCCCCCATAGT
N-cadherin	TGTGCAAGATGGGATTTCCTGA
	GAGCAGAGGTTGTCCTCCAT
β-catenin	GGGTTGATTCGCAATCTCGC
	CCTCCACAAACTGCTGTTGC
GAPDH	GAGGGTAGTGAAGGCTGCTG
	CACAACACGGTTGCTGTATC

## References

[1] Stanton P.G. (2016). Regulation of the blood-testis barrier. Semin. Cell Dev. Biol..

[2] Su L., Mruk D.D., Cheng C.Y. (2011). Drug transporters, the blood-testis barrier, and spermatogenesis. J. Endocrinol..

[3] Xiao Y., Zhang J., Guan Y., Guan Y., Wang M., Liu D., Xiong S., Li J., Yu X. (2025). Research progress on Sertoli cell secretion during spermatogenesis. Front. Endocrinol..

[4] Feng S., Wen H., Liu K., Xiong M., Li J., Gui Y., Lv C., Zhang J., Ma X., Wang X. (2023). hnRNPH1 establishes Sertoli-germ cell crosstalk through cooperation with PTBP1 and AR, and is essential for male fertility in mice. Development.

[5] Lai F., Wang H., Zhao X., Yang K., Cai L., Hu M., Lin L., Xia X., Li W., Cheng H. (2023). RNF20 is required for male fertility through regulation of H2B ubiquitination in the Sertoli cells. Cell Biosci..

[6] Wu D., Zhang K., Khan F.A., Pandupuspitasari N.S., Liang W., Huang C., Sun F. (2022). Smtnl2 regulates apoptotic germ cell clearance and lactate metabolism in mouse Sertoli cells. Mol. Cell. Endocrinol..

[7] Rougerie P., Largeteau Q., Megrelis L., Carrette F., Lejeune T., Toffali L., Delon J. (2013). FAM65B is a new transcriptional target of FOXO1 that regulates RhoA signaling for T lymphocyte migration. J. Immunol..

[8] Yoon S., Molloy M.J., Wu M.P., Cowan D.B., Gussoni E. (2007). C6ORF32 is upregulated during muscle cell differentiation and induces the formation of cellular filopodia. Dev. Biol..

[9] Froehlich J., Versapuech M., Megrelis L., Largeteau Q., Meunier S., Tanchot C., Bismuth G., Delon J., Mangeney M. (2016). FAM65B controls the proliferation of transformed and primary T cells. Oncotarget.

[10] Gao K., Tang W., Li Y., Zhang P., Wang D., Yu L., Wu D. (2015). Front-signal-dependent accumulation of the RHOA inhibitor FAM65B at leading edges polarizes neutrophils. J. Cell Sci..

[11] Balasubramanian A., Kawahara G., Gupta V.A., Rozkalne A., Beauvais A., Kunkel L.M., Gussoni E. (2014). FAM65B is important for formation of the HDAC6-dysferlin protein complex during myogenic cell differentiation. FASEB J..

[12] Del Re D.P., Miyamoto S., Brown J.H. (2008). Focal adhesion kinase as a RhoA-activable signaling scaffold mediating Akt activation and cardiomyocyte protection. J. Biol. Chem..

[13] Papakonstanti E.A., Ridley A.J., Vanhaesebroeck B. (2007). The p110 delta isoform of PI 3-kinase negatively controls RhoA and PTEN. EMBO J..

[14] Xu H., Liu P., Liang L., Danesh F.R., Yang X., Ye Y., Zhan Z., Yu X., Peng H., Sun L. (2006). RhoA-mediated, tumor necrosis factor α-induced activation of NF-κB in rheumatoid synoviocytes: Inhibitory effect of simvastatin. Arthritis Rheum..

[15] Polk W.W., Ellis C.A., Kushleika J.V., Simmonds P.L., Woods J.S. (2007). RhoA regulation of NF-κB activation is mediated by COX-2-dependent feedback inhibition of IKK in kidney epithelial cells. Am. J. Physiol. Cell Physiol..

[16] Zhu R., Wang J., Feng T., Hu X., Jiang C., Wang X., Li K., Sang Y., Hua Y., Sun H. (2019). The alteration of RhoA geranylgeranylation and Ras farnesylation breaks the integrity of the blood-testis barrier and results in hypospermatogenesis. Cell Death Dis..

[17] Liu Y., Fan J., Yan Y., Dang X., Zhao R., Xu Y., Ding Z. (2020). JMY expression by Sertoli cells contributes to mediating spermatogenesis in mice. FEBS J..

[18] Wang J., Bao B., Feng J., Zhao Q., Dai H., Meng F., Deng S., Wang B., Li H. (2021). Effects of Diabetes Mellitus on Sperm Quality in the Db/Db Mouse Model and the Role of the FoxO1 Pathway. Med. Sci. Monit..

[19] Johnson L., Thompson D.L., Varner D.D. (2007). Role of Sertoli cell number and function on regulation of spermatogenesis. Anim. Reprod. Sci..

[20] Chojnacka K., Zarzycka M., Mruk D.D. (2016). Biology of the Sertoli Cell in the Fetal, Pubertal, and Adult Mammalian Testis. Results Probl. Cell Differ..

[21] Lucas T.F.G., Lazari M.F.M., Porto C.S. (2013). Differential role of the estrogen receptors ESR1 and ESR2 on the regulation of proteins involved with proliferation and differentiation of Sertoli cells from 15-day-old rats. Mol. Cell. Endocrinol..

[22] Li J., Sun B., Wang D., Liu Y., Qi J., Nie X., Liang S. (2021). Melatonin ameliorates cypermethrin-induced impairments by regulating oxidative stress, DNA damage and apoptosis in porcine Sertoli cells. Theriogenology.

[23] Zhao Y., Chen M., Wang J., Cui J., Zhang H., Li X., Li J. (2022). Connexin-43 is a promising target for lycopene preventing phthalate-induced spermatogenic disorders. J. Adv. Res..

[24] Umar S., Wang Y., Morris A.P., Sellin J.H. (2007). Dual alterations in casein kinase I-epsilon and GSK-3beta modulate beta-catenin stability in hyperproliferating colonic epithelia. Am. J. Physiol. Gastrointest. Liver Physiol..

[25] Lei W.L., Li Y.Y., Meng T.G., Ning Y., Sun S.M., Zhang C.H., Gui Y., Wang Z.B., Qian W.P., Sun Q.Y. (2021). Specific deletion of protein phosphatase 6 catalytic subunit in Sertoli cells leads to disruption of spermatogenesis. Cell Death Dis..

[26] Liu H., Zeng X., Ma Y., Chen X., Losiewicz M.D., Du X., Tian Z., Zhang S., Shi L., Zhang H. (2022). Long-term exposure to low concentrations of MC-LR induces blood-testis barrier damage through the RhoA/ROCK pathway. Ecotoxicol. Environ. Saf..

[27] Wu Y., Duan P., Wen Y., Zhang J., Wang X., Dong J., Yuan S. (2022). UHRF1 establishes crosstalk between somatic and germ cells in male reproduction. Cell Death Dis..

[28] Mruk D.D., Cheng C.Y. (2011). An in vitro system to study Sertoli cell blood-testis barrier dynamics. Methods Mol. Biol..

